# QPLOT: A Quality Assessment Tool for Next Generation Sequencing Data

**DOI:** 10.1155/2013/865181

**Published:** 2013-11-11

**Authors:** Bingshan Li, Xiaowei Zhan, Mary-Kate Wing, Paul Anderson, Hyun Min Kang, Goncalo R. Abecasis

**Affiliations:** ^1^Department of Physiology and Biophysics, Center for Human Genetics Research, Vanderbilt University, Nashville, TN 37232, USA; ^2^Department of Biostatistics, Center for Statistical Genetics, University of Michigan, Ann Arbor, MI 48109, USA

## Abstract

*Background.* Next generation sequencing (NGS) is being widely used to identify genetic variants associated with human disease. Although the approach is cost effective, the underlying data is susceptible to many types of error. Importantly, since NGS technologies and protocols are rapidly evolving, with constantly changing steps ranging from sample preparation to data processing software updates, it is important to enable researchers to routinely assess the quality of sequencing and alignment data prior to downstream analyses. *Results.* Here we describe QPLOT, an automated tool that can facilitate the quality assessment of sequencing run performance. Taking standard sequence alignments as input, QPLOT generates a series of diagnostic metrics summarizing run quality and produces convenient graphical summaries for these metrics. QPLOT is computationally efficient, generates webpages for interactive exploration of detailed results, and can handle the joint output of many sequencing runs. *Conclusion.* QPLOT is an automated tool that facilitates assessment of sequence run quality. We routinely apply QPLOT to ensure quick detection of diagnostic of sequencing run problems. We hope that QPLOT will be useful to the community as well.

## 1. Introduction

Next generation sequencing (NGS) is a revolutionary technology for biomedical research and is being deployed in a variety of applications, ranging from the identification of rare variants, *de novo* mutations, and somatic mutations in human disease studies to assessments of transcriptome and epigenome states in cultured cells. Since NGS provides more complete results than traditional array technologies and is rapidly decreasing in cost, it is becoming more widely used for genomics studies. Whole exome sequencing, which is the targeted sequencing of the entire collection of protein coding regions in the genome, has already led to great advances in Mendelian disorder genetics [1, 2], complex traits [3, 4],and cancer genomics [5, 6]. The 1000 Genomes Project [7, 8] is leading an effort to provide a comprehensive catalog of human variation across the world through whole genome sequencing. Several underway studies are now deploying whole genome and whole exome sequencing to study large collections of human disease samples.

The success of NGS studies depends on appropriately understanding the quality of underlying data. However, unlike traditional array platforms, analysis of sequencing data is much more complex, making real time monitoring of data quality more challenging. NGS technologies and associated set of protocols are constantly evolving, and updates to several different components of the process (including, e.g., software, sample preparation, and/or reagents) can result in important and sometimes unexpected changes in data quality. We believe that the ability to generate automated visual summaries that help identify common problems is critical. To achieve this, we developed QPLOT, a tool for quick quality assessment in NGS data. QPLOT calculates and graphs summary statistics describing sequence and alignment quality. Data quality is assessed both through reported base quality scores and empirically obtained metrics by comparing aligned bases to the reference genome. In this way, it is possible to track the number of high quality bases along the length of a read (to choose a read length that maximizes the yield of high quality bases and compare run quality over time) or identify the presence of adaptor sequence and other problems in alignment (these can result in high empirical mismatch rates near the ends of RNA-sequencing reads, due to difficulties in correctly placing splice junctions—a problem that can be ameliorated by excluding these bases from variant calling and RNA editing analyses *after* alignment). We constantly interact with our sequencing core and other collaborators generating sequence data to improve QPLOT and facilitate efforts to drive up the quality of next generation sequence data.

QPLOT differs from tools that only inspect unaligned sequence reads (such as FastQC [9] and SolexaQA [10]), because it can identify common problems in alignment and provide diagnostic descriptions of read mapping. For example, it generates empirically calibrated base quality scores and insert size distributions, two features that have substantial impact on variant calling and other downstream analyses. QPLOT also tries to improve packages designed specifically for handling aligned data (such as SAMStat [11] and Picard [12]) through its computational efficiency (QPLOT can sample regions of the genome randomly so as to rapidly evaluate very large alignments) and its ability to handle many samples (which helps to identify batch effects and other transient data processing problems). Importantly we note that genome-wide summary statistics can be extrapolated based on randomly sampled regions with little loss of accuracy. When the number of input files is very large, QPLOT can generate XML and text files with raw summary data and an interactive webpage that allows users to explore available quality metrics and graphs. XML and text output can be conveniently stored in a tracking database. In addition to graphical representation, key features are also summarized to generate a concise representation of the quality measurement (e.g., a mean squared difference is used to summarize concordance of empirical and reported base quality scores, and the impact of GC content is summarized in a similar fashion based on the deviation of the depth for each GC bin from uniform coverage).

## 2. Materials and Methods

QPLOT is implemented in C++ and invokes R to generate figures. Available statistics include summaries of base quality, both overall and along each position in a read, comparisons of reported and empirical quality base scores, summaries of insert size for paired end libraries, global evaluations of coverage as well as more detailed evaluations of coverage as a function of GC content, and the regions targeted for enrichment. Empirical base scores are calculated as Phred scaled mismatch rates, that is, −10 × log_10_ (number of matches/(number of matches + number of mismatches)), where number of matches and number of mismatches are the counts of aligned sequence bases that are concordant or discordant with the expected base in the reference genome, respectively, excluding known variant sites; these mismatches are dominated by genuine sequencing errors and provide a basis for base quality recalibration. To describe potential GC bias in sequencing runs, we calculate the mean depth of coverage for each GC content bin (0–100 representing 0–100% GC composition) for a series of windows along the genome (or, in the case of targeted sequencing experiments, within targeted regions). After normalization by the expected depth based on total mapped reads, the normalized depth for each GC content bin reflects biases of each experiment and can be compared with sequenced samples. Details of other summary statistics are available on the QPLOT website (http://genome.sph.umich.edu/wiki/QPLOT). QPLOT can be run as a stand-alone tool or incorporated into automated data processing pipelines.

## 3. Results and Discussion

**Figure 1:**
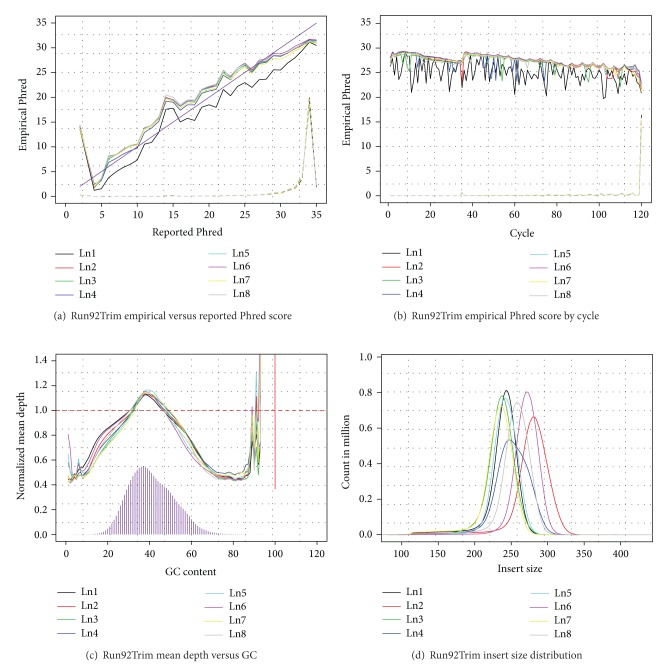
A subset of figures generated by QPLOT on an Illumina run. (a) Empirical base quality scores versus the scores stored in the BAM files. (b) Empirical base quality scores by cycles. (c) Bias of depth by GC content. (d) Insert size distribution.

We regularly use QPLOT in our sequencing projects including whole genome sequencing, RNA-seq, and targeted sequencing. Results for one Illumina run in a whole genome low pass sequencing study are shown in Figure 1. In this run the reported base quality scores deviate from empirically assessed quality, indicating that base quality recalibration is recommended (Figure 1(a)). As expected, empirical base quality scores decrease with increasing position along reads (Figure 1(b)), which is typical of Illumina sequencing. However at position 36 empirical quality scores appear to increase, an artifact of the −q 15 option used in BWA [13] when mapping these data. The −q 15 option trims portions of reads with base quality <15, but always leaves at least 36 bases in each read (in our experience, this option increases the fraction of mapped reads and the number of mapped high quality bases). In this run, sequences with very high or very low GC content are underrepresented (below 1 in the relative depth curve, Figure 1(c)). Assessment of paired reads shows a distribution of insert sizes with peaks ranging from ~240 bp to ~300 bp (Figure 1(d)). In this case, since reads are 120 bases long, many paired reads overlap (particularly in lanes 1, 3, 5, and 7);these overlaps, if ignored, can result in PCR artifacts that look like sequence variants—suggesting that the protocol might be tweaked to increase library insert sizes. When we compared metrics generated by evaluating the complete data and those extrapolated from random 5 Mb segments of the genome, the two sets of summary statistics were remarkably similar (see QPLOT webpage, e.g.), but computing time was reduced from 38 minutes to 13 minutes.

**Figure 2:**
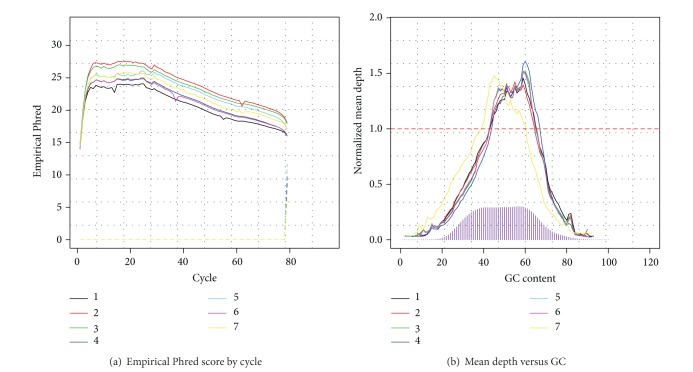
Exemplar diagnosis plots of RNA-sequencing data. (a) Empirical base quality scores by cycles. (b) Differential GC biases across multiple samples.

In a second example, Figure 2 summarizes the results of an RNA-sequencing run. Here, empirical base quality scores are unexpectedly low near the beginning of each read (Figure 2(a)). When we remapped all reads after trimming the first several bases, the same pattern was repeated, suggesting that the observation is not due to high sequencing error rates or residual adapter sequences (trimming and remapping usually solve problems with residual adapter sequences, in our experience). Instead, the observation is the result of alignment artifacts when exon boundaries fall near the beginning or end of reads, a common problem in RNA-sequencing analyses. To avoid artifacts in downstream analyses, we suggest trimming the beginning and end bases of each read *after* mapping.  Figure 2(b) shows that lane 7 has a GC content pattern that is dramatically different from the others, recommending great caution before comparing gene expression levels estimated for that sample and the others [14].

## 4. Conclusions

NGS has revolutionized the way genomics and biomedical studies are conducted. However the technologies are still rapidly evolving, and analysis of NGS data is challenging. Simple and convenient tools are important to help monitor data production and processing. Here we describe QPLOT, a computationally efficient tool that we hope will be helpful in quality assessment and diagnosis of NGS performance. We hope that information conveyed in these plots and statistics will facilitate the understanding of sequencing data to enable improved downstream processing and constant quality improvements.
